# Hierarchical Differentiation of Myeloid Progenitors Is Encoded in the Transcription Factor Network

**DOI:** 10.1371/journal.pone.0022649

**Published:** 2011-08-10

**Authors:** Jan Krumsiek, Carsten Marr, Timm Schroeder, Fabian J. Theis

**Affiliations:** 1 Institute of Bioinformatics and Systems Biology, Helmholtz Zentrum München, München, Germany; 2 Institute of Stem Cell Research, Helmholtz Zentrum München, München, Germany; 3 Department of Mathematics, Technische Universität München, München, Germany; Centro Cardiologico Monzino, Italy

## Abstract

Hematopoiesis is an ideal model system for stem cell biology with advanced experimental access. A systems view on the interactions of core transcription factors is important for understanding differentiation mechanisms and dynamics. In this manuscript, we construct a Boolean network to model myeloid differentiation, specifically from common myeloid progenitors to megakaryocytes, erythrocytes, granulocytes and monocytes. By interpreting the hematopoietic literature and translating experimental evidence into Boolean rules, we implement binary dynamics on the resulting 11-factor regulatory network. Our network contains interesting functional modules and a concatenation of mutual antagonistic pairs. The state space of our model is a hierarchical, acyclic graph, typifying the principles of myeloid differentiation. We observe excellent agreement between the steady states of our model and microarray expression profiles of two different studies. Moreover, perturbations of the network topology correctly reproduce reported knockout phenotypes *in silico*. We predict previously uncharacterized regulatory interactions and alterations of the differentiation process, and line out reprogramming strategies.

## Introduction

**Figure 1:**
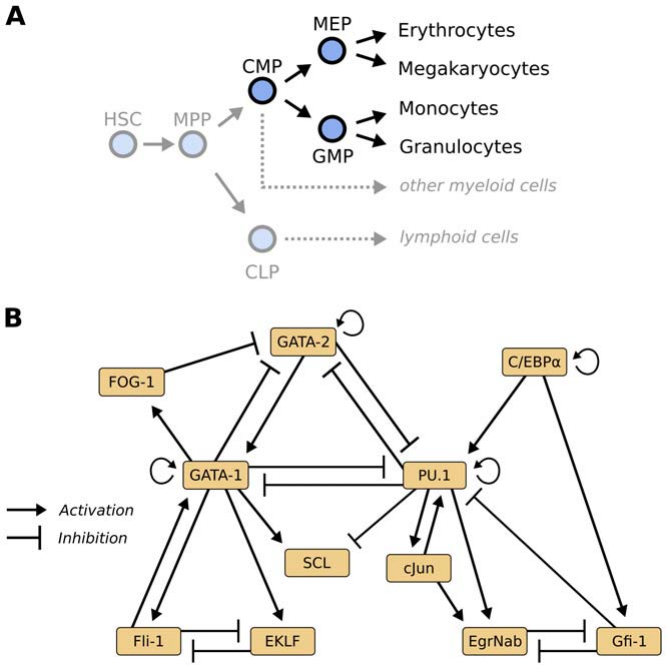
A regulatory model of myeloid differentiation. (**A**) Hematopoietic stem cell (HSC) differentiation consists of a series of switch-like decisions. We here focus on differentiation into four myeloid cell types and omit other myeloid cells and the lymphoid branch (greyed out). For a detailed discussion of the different progenitor cell types, we refer the reader to [1]. Abbreviations: MPP, multipotent progenitor; CMP, common myeloid progenitors; MEP, megakaryocyte-erythrocyte progenitor; GMP, granulocyte-monocyte progenitor; CLP, common lymphoid progenitor. (**B**) Literature-derived gene regulatory network for 11 myeloid players previously reported to be pivotal for the lineage decisions in myeloid differentiation (compare Text S1). Note that this visualization does not contain explicit Boolean update rules. Specifically, it is not apparent from the graph visualization alone whether multiple regulatory inputs are combined using AND or OR logic, which can make substantial differences for the resulting Boolean dynamics.

Hematopoiesis – a system with a well-known biological background and advanced experimental access – is considered as a paradigm for stem cell biology [1]. From a single cell type, the hematopoietic stem cell (HSC), all mature blood cells emerge through a hierarchical series of lineage decisions via different progenitor cells [2]. Thus, hematopoiesis is often depicted as a hierarchical differentiation tree, with a HSC at the root and the mature blood cells as the leaves (Figure 1A). One of the intermediate cellular states is the common myeloid progenitor (CMP) [3]. CMPs can proliferate and differentiate into megakaryocyte-erythrocyte (MegE) progenitors and granulocyte-monocyte (GM) progenitors, which further give rise to megakaryocytes, erythrocytes, granulocytes, monocytes and others. In the past 20 years, a number of regulatory interactions between important transcription factors, governing the differentiation process, have been experimentally unraveled (for a review, see [4]).

Certain aspects of hematopoiesis have previously been described with mathematical models incorporating detailed kinetic rate laws (cf. [5]–[8].) Often however, only qualitative information on the regulatory interactions (like ‘gene A activates gene B’) is available. In these cases, a modeling framework that abstracts from actual compound concentration is needed. An early model of binary gene regulatory networks shaping differentiation was published by Kauffman [9] in 1969. Following that approach, Boolean networks have been applied successfully to the description of, e.g., plant morphogenesis [10], [11], the yeast cell-cycle [12], [13], gene expression patterning in Drosophila melanogaster [14] and the developing mouse brain [15]. While it is obvious that this methodology cannot account for continuous changes of concentrations or the exact timing of regulatory events, it allows to explore the functional capabilities of the system without knowledge of any kinetic parameters. This is especially favorable if the studied system contains more than just two or three factors [16].

A Boolean network is discrete in space and time, each node 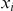 is either ‘on’ or ‘off’. The state of a node is updated depending on the current states of all nodes in the network: 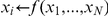. The respective update function 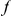 can be conveniently defined by Boolean equations and logical operators. For instance 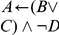 represents a regulatory interaction where A will be ‘on’ if and only if at least one of the activators B and C is present and the inhibitor D is absent. A Boolean system can be updated *synchronously*, that is all factors are updated simultaneously. This scenario leads to a fully deterministic model, where each Boolean state has at most one follow-up state, and only a single trajectory through the system exists from any given initial state. In contrast, when updating *fully asynchronously* [17], the state value of only a single factor is changed during each update. In this scenario, multiple trajectories through the system are possible. Since we here attempt to describe the decision-making processes in differentiating myeloid progenitor cells, a modeling approach that allows for multiple trajectories through the state space is desirable. By connecting all states with their follow-up states we compute the *state transition graph*, which represents the complete dynamic potential of the underlying network. States with no consecutive state represent end-points of the system. These so-called *steady state attractors* then correspond to the mature cell types in the biological context of differentiation.

From a thorough examination of the literature on myeloid differentiation in mouse, we devised a network with Boolean regulatory logic of transcription factors, from now on called the *players* of the system. We studied the topology of the network and analyzed the kinetics of small regulatory modules. Under asynchronous updating, the regulatory network induces an acyclic, hierarchical state space, whose different branches can be directly attributed to known biological cell states. For validation, we explicitly compared the Boolean states of the attractors with gene expression profiles of differentiating and mature myeloid blood cells. We confirm the the predictive power of our model by *in silico* perturbations of players and interactions and compare the results with known molecular phenotypes.

## Results

### Model construction

The scope of our model is the differentiation of common myeloid progenitors (CMPs) into erythrocytes, megakaryocytes, granulocytes and monocytes (see Figure 1A). We disregard earlier hematopoietic stages (like the lineage switch between the lymphoid and the myeloid lineage) and other blood cell lineages (like the differentiation of mast cells or neutrophils from granulocytes). From recent reviews and overview papers [1], [4], [18],[19] we assembled a set of 11 central myeloid transcription factors, known to orchestrate the respective differentiation decisions. The set comprises early hematopoietic factors (GATA-2, C/EBP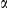), intermediate factors (GATA-1, PU.1) as well as late, secondary fate determinants and cofactors (EKLF, Fli-1, FOG-1, SCL, Gfi-1, cJun, EgrNab). The latter factor, EgrNab, represents an integration of Egr-1, Egr-2 and Nab-2. While the three players play distinct roles during other hematopoietic processes, Laslo et al. [20] demonstrated highly correlated expression patterns as well as similar functional roles in the context of myeloid differentiation. The roles of all 11 factors and their respective gene products have been determined by knockout, over-expression and expression profiling studies (for an overview, see [18]). In addition, many of the genes included in the model are known to be involved in malignant cell transformations during hematopoiesis [21], [22]. Hematopoietic players which act only in monopotent lineages or non-myeloid hematopoiesis were excluded from our model. In the following, we examplarily discuss five such cases. (i) NF-E2 is regulated by GATA-1 and SCL, but specifically important for megakaryocytic development [23]–[25]. (ii) Similarly, IRF8 is required for macrophage [26] and B-cell [27] differentiation and was thus excluded. (iii) While C/EBP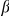 is known to rescue targeted disruption of C/EBP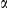, its primary physiological role lies in macrophage differentiation [28]. (iv) The erythroid transcription factor Gfi-1b is induced by GATA-1 [29] and required in both erythrogenesis and megakaryogenesis [30], and is thus not involved in the megakaryocyte vs. erythrocyte lineage decision. (v) RUNX1 is an early transcription factor required in HSCs [31] which is reused later in the differentiation process for the megakaryocyte lineage [32]. To the best of our knowledge, no direct role in myeloid lineage decision has been described for RUNX1.

We generated a qualitative interaction model of myeloid differentiation by investigating potential regulatory interactions proposed by the Bibliosphere [33] text-mining tool (Figure 1B). For the derivation of concrete Boolean update rules, we manually interpreted the respective papers and the biochemical interactions proposed therein. For example, GATA-2 activates its own promoter, and is synergistically inhibited by GATA-1 and FOG-1. As both players are required to exhibit the full inhibitory effect, we combined them using an AND logic in the Boolean update rule. For the regulation of PU.1, both GATA-1 and GATA-2 independently suppress the PU.1 promoter, and thus we employed an OR logic for this case. Again, we paid special attention to incorporate only those interactions which have been reported for adult murine cells during myeloid differentiation. The list of all update rules we derived for the 11 players along with short justifications and references is given in Table 1. For a detailed discussion of the role of each factor as well as its regulatory interactions, we refer the reader to Text S1. Note that we propose the inhibition of C/EBP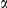 by an erythroid factor (see discussion below).

**Table 1:** List of transcription factors in our myeloid differentiation model.

Factor	Boolean update rule	Comments and References
GATA-2	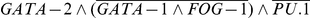	Early MegE factor [81]; autoregulatory activation, which is synergistically inhibited by GATA-1 and FOG-1 [81], [82]; inhibited by PU.1 [49], [83]
GATA-1	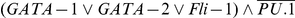	Central MegE factor [54]; activated by GATA-2 during early hematopoiesis [81]; autoregulatory activation [84]–[86]; stimulated by downstream factor Fli-1 [87]; inhibited by PU.1 [49], [83]
FOG-1	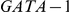	GATA-1 cofactor [70], [71]; activated by GATA-1 [59]
EKLF	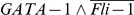	Erythroid factor activated by GATA-1 [88]; antagonized by Fli-1 [87]
Fli-1	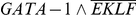	Megakaryocytic factor activated by GATA-1 [89]; antagonized by EKLF [87]
SCL	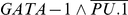	Central hematopoietic player, but specifically involved in MegE differentiation [1], [36]; activated by GATA-1 during erythroid differentiation [90]; inhibited by PU.1 [91]
C/EBP 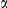	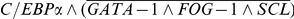	Early GM factor [45]; no known upstream regulatory player, we assume an inhibitory influence from MegE-specific factors due to downregulation [18]; questionable FOG-1 inhibition proposed in [92] ^1^
PU.1	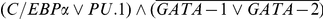	Central GM factor [55]; activated by C/EBP 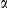 during GM development [45], [46]; autoregulatory activation [93], [94]; mutual inhibition with GATA factors [49], [83]
cJun	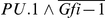	PU.1 expression required for its cofactor cJun [95]; Gfi-1 antagonizes PU.1 transcriptional activity [47]
EgrNab	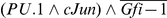	Integrated monocytic factor (Egr-1, Egr-2, Nab-2) according to [20]; activated by PU.1+cofactor cJun [20]; mutual antagonism with Gfi-1 [20]
Gfi-1	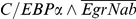	Granulocytic factor [79]; activated by C/EBP 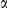 , antagonized by EgrNab [20]

The table displays the Boolean update rules for all factors and brief justifications along with references to the respective publications. Notation: 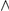 = AND, 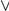 = OR, 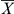 = not X.

It is important to understand that transcription factors are commonly reused in varying contexts during stem cell differentiation processes. For instance, GATA-2, SCL, Fli-1 and Gfi-1 are also known to play important roles in early hematopoietic stem cells [8], [31], [34]–[36]. The counter-antagonists PU.1 and GATA-1 synergize in the development of the eosinophil lineage [37], whereas the mutual inhibition between PU.1 and GATA-2 is absent in mast cell progenitors [38]. Here, we focused on the differentiation of CMPs to erythrocytes, megakaryocytes, granulocytes and monocytes and deliberately ignored interactions and players important in earlier and later stages of hematopoietic differentiation process (see Figure 1A).

### Local dynamics

Our regulatory network comprises 11 players and 28 interactions (Figure 1B). It is composed of a set of well-known regulatory motifs, each of which contributes a specific functionality to the overall system dynamics (see [39] for an extensive review). To get insights into local dynamic capabilities of the system, we study central functional modules in the network.

First, we observe four mutually inhibitory pairs of genes in our network (PU.1 vs. GATA-1, PU.1 vs. GATA-2, Gfi-1 vs. EgrNab, EKLF vs. Fli-1). This regulatory module is known to generate a toggle-switch behavior of the involved factors (cf. e.g. [40]): Only one of the two genes can be fully active at any given time, and thus the switch induces stable decisions between antagonistic lineages. Note that mutual inhibitory switches belong to the class of positive feedback loops, which are structurally required for the occurrence of multistationarity in dynamical systems [41], [42]. We observe additional positive feedback loops, either in the form of direct autoregulation (GATA-2, GATA-1, C/EBP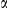, PU.1), or via indirect mechanisms (as seen between PU.1 and cJun or GATA-1 and Fli-1). Such activatory positive feedbacks could be means to stabilize lineage-specific expression patterns or, in addition, can have specific roles in the interplay of certain factors (see e.g. GATA-2/GATA-1/FOG-1 module below).

**Figure 2:**
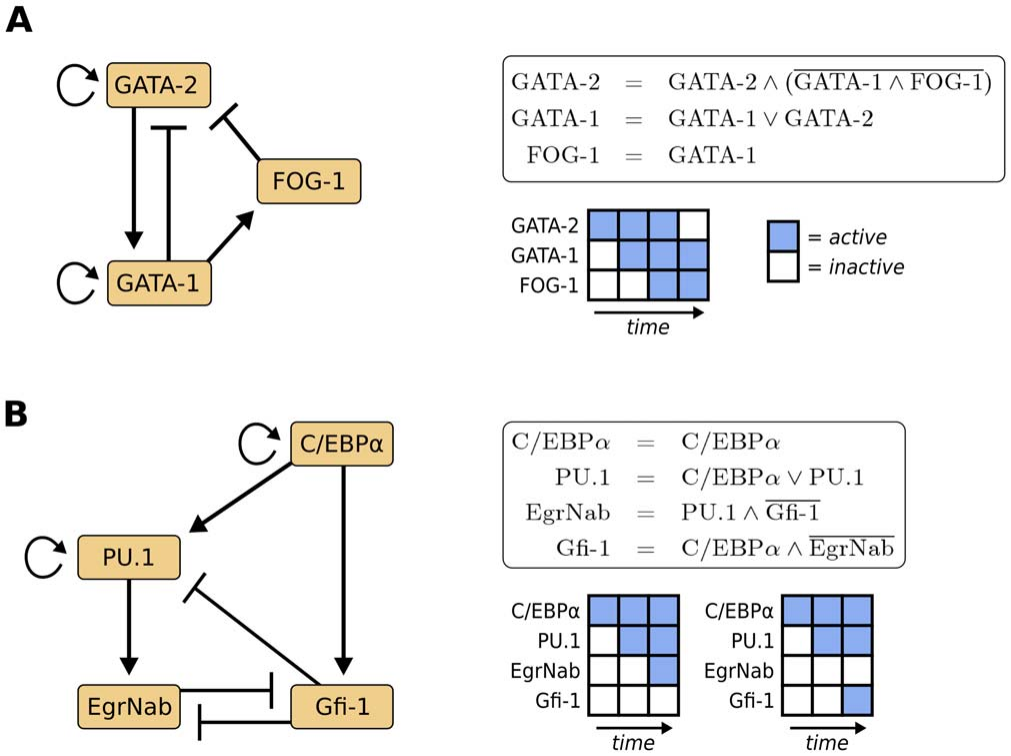
Functional modules in the network. (**A**) The GATA-2, GATA-1, FOG-1 regulatory circuit consists of a coherent inhibitory feed-forward loop (GATA-1 and FOG-1 towards GATA-2), an autoregulation (by GATA-1) and a negative feedback (GATA-2 onto itself). The corresponding Boolean update rules and a schematic of the Boolean dynamics is shown on the right, demonstrating how the system is pushed towards maturation. GATA-2 activates its downstream target GATA-1, which synergizes with its cofactor FOG-1 to downregulate GATA-2. Due to the autoregulatory loop, GATA-1 can sustain its expression after its upstream regulator is inhibited. (**B**) Asymmetric activation of EgrNab and Gfi-1. The gene switch is driven by an upstream feed-forward loop around C/EBP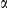 and PU.1. The Boolean update rules between the four players and two possible system trajectories are shown on the right. C/EBP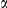 initially activates PU.1, but can also upregulate its antagonist Gfi-1 which then inhibits the PU.1 target EgrNab. Note that the two stable states - one where EgrNab is finally activated and one where Gfi-1 is activated - are mutually exclusive.

A remarkable dynamic behavior is constituted by GATA-2, GATA-1 and FOG-1, which are connected through three nested regulatory modules: (i) a coherent, inhibitory feed-forward loop [43] from GATA-1 to GATA-2, (ii) GATA-1 positive autoregulation and (iii) the negative feedback loop of GATA-2 through the other two factors (Figure 2A). This circuit enforces a time-delayed switch from GATA-2 expression to GATA-1 expression. GATA-2 induces the expression of GATA-1, which first activates its cofactor FOG-1, and then downregulates GATA-2 cooperatively with FOG-1. This synergy causes a delayed downregulation of GATA-2 via the feed-forward loop. Consistently, GATA-2 has been identified as a gatekeeper for immaturity in hematopoietic progenitor cells [44]. The negative feedback does not lead to an oscillatory behavior due to the GATA-1 autoregulation, which sustains its own expression after the upstream activator GATA-2 is depleted.

Interestingly, while GATA-1 symmetrically activates its downstream targets, we observe an asymmetrical structure for the activation of Gfi-1 and EgrNab (Figure 2B). Apart from the mutually inhibitory switch between Gfi-1 and EgrNab, there is an incoherent feed-forward loop [43] formed by C/EBP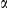, Gfi-1 and PU.1. C/EBP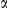 initially activates PU.1 [45], [46], but then turns into its indirect antagonist by activating Gfi-1. This transcription factor is known to inhibit the transactivation activity of PU.1, while PU.1 expression levels remain unchanged (cf. Text S1 for detailed evidence). This provides an elegant possibility for a time-delayed mechanism, where PU.1's downstream activity will be inhibited unless it activates its downstream target EgrNab before Gfi-1 is present. Note that the inhibition of EgrNab by Gfi-1, proposed as a direct interaction by Laslo et al. [20], could also be established by the repression of PU.1 activity through Gfi-1 [47].

### Systems dynamics

The two modules investigated in the previous section comprised the core dynamic features of the MegE and GM lineage decision processes, respectively. We next sought to investigate the dynamic potential of our regulatory system as a whole. In order to impose differential dynamics for all players, each node in the network requires at least one upstream regulatory factor. To the best of our knowledge, no such regulator is known for the early player C/EBP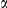 (see Figure 1B). It is well-known, however, that C/EBP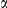 is strongly downregulated during MegE differentiation (cf. e.g. [18]). From our player list, this downregulation can only be achieved by inhibitory influences of one of the MegE players GATA-1, FOG-1, SCL, or a synergistic action between those three (Table 1). This inhibitory influence could be direct, i.e. by transcription factor binding, or mediated by a third, yet unknown player.

**Figure 3:**
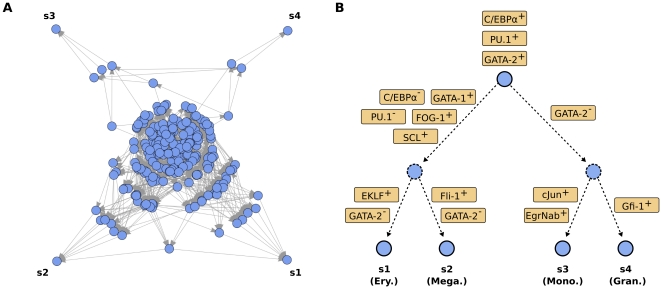
Systems dynamics of the myeloid differentiation model. (**A**) State-transition graph of the Boolean model with dynamical trajectories. Each node represents a Boolean state of the system where each player is either ‘on’ or ‘off’. Each edge stands for a transition between two states induced by the application of a single Boolean update rule. The shown subgraph is calculated from an central early hematopoietic state and comprises 232 nodes with 789 links. The visualization emphasizes the existence of four attractors reachable from the early state, and the hierarchical structuring of the state space with two pairs of attractors (s1/s2 and s3/s4, respectively) that share a common attractor basin. The distance of a state from the attractor in the graph corresponds to the number of necessary update steps. (**B**) Interpretation of the state space in the context of myeloid differentiation. We observe a hierarchical partitioning with subsequent splits between the GM and MegE lineages, followed by splits of the granulocyte and monocyte lineages, and the erythrocyte and megakaryocyte lineages, respectively. Arrows in the diagram represent expression changes on the respective branch of the differentiation tree.

A Boolean network translates into a state-transition graph, where each node represents a specific state of the system and each link is a transition between two states. These links arise from the Boolean update rules and thus represent the dynamic potential of the system. Our 11 player model gives rise to a 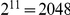 node state-transition graph, which contains five non-zero attractors (i.e. steady states of the system). In the following, we focus on the dynamics initiated by an early, unstable undifferentiated state, where only GATA-2, C/EBP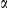, and PU.1 are active (cf. [48], [49] for experimental evidence on the early expression state). The subgraph of all nodes downstream of this early state comprises 232 nodes and 789 links (see Figure 3A) and has a number of salient properties: (i) four of the five non-trivial attractors of the system (denoted as s1 to s4 in Figure 3A) can be reached from the early state, (ii) the graph is acyclic, forcing the system to move from the early state towards one of the attractors, and (iii) it exhibits a hierarchical partitioning into non-overlapping subparts.

We evaluated the extend of hierarchicity by calculating the attractor basin overlaps of the reachable steady states s1–s4. That is, how many of the states that can lead to a given attractor may also lead to another attractor? We identify two pairs of attractors with a respective overlap of over 50%. The basins of the first pair of attractors (s1 and s2 in Figure 3A) are characterized by the activity of the factors GATA-1 and FOG-1, early activity of GATA-2 and absence of GM-specific factors in general. Vice versa, states that lead to the second pair of attractors (s3 and s4 in Figure 3A) exhibit activated C/EBP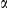, PU.1 and cJun while all MegE factors are deactivated. From the known roles of the secondary lineage-specific factors Gfi-1, EgrNab, EKLF and Fli-1 (see, e.g. [1]) we assign each of the attractors to one of the four myeloid lineages: erythrocytes (s1), megakaryocytes (s2), monocytes (s3) and granulocytes (s4), respectively, see Figure 3B.

While the subgraph downstream of our defined early state is certainly of most relevance in the context of myeloid differentiation, we also analyzed the remaining states upstream of the early state and the supposedly unphysiological fifth stable attractor (with PU.1, cJun and EgrNab active, see above). Any state that reaches the early state also requires GATA-2, C/EBP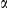, and PU.1 to be active. The basin of the early state contains all those states that show unstable expression of downstream factors, e.g. activity of EKLF without presence of GATA-1. Such states cannot be considered physiologically relevant, and the early state supposedly represents the true starting point of the modeled biological system. The fifth steady state, on the other hand, can be reached if PU.1 is active and either GATA-1 is expressed or C/EBP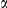 is absent. That is, the state can be reached whenever it is possible to sustain PU.1 expression while C/EBP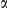 vanishes (which is not the case from the early state used in our analysis).

### Evaluation of the state space attractors with mRNA expression data

**Figure 4:**
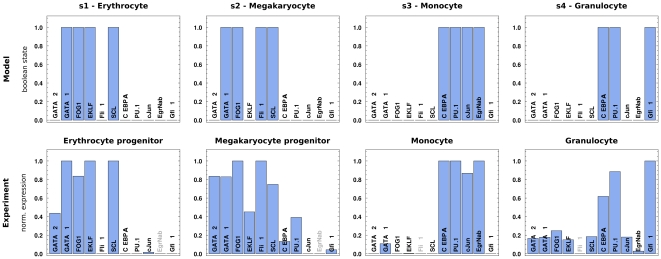
Comparison of Boolean states (top) with normalized mRNA expression profiles (bottom) for the 11 players of our model (see text for a detailed discussion). We observe a good agreement between model prediction and measured mRNA expression. Note that we excluded non-differentially expressed genes with a maximum fold change smaller than 2 in all samples of the respective study (EgrNab in Pronk et al. [51] and Fli-1 in Chambers et al. [50], greyed out). For a discussion of the mismatch between prediction and data for GATA-2, see main text.

In order to assess whether the four attractors indeed correspond to the respective cellular fates, we compared the Boolean states with mRNA expression data from two independent microarray experiments (see Methods for a detailed description). Maturated granulocyte and monocyte-specific expression data were taken from Chambers et al. [50], megakaryocyte and erythrocyte progenitor cell profiles from Pronk et al. [51]. Note that the Chambers dataset contains a maturated erythrocyte sample which is in general agreement with the erythrocyte progenitors from Pronk et al. (see below and Text S2). The comparison of experimental expression data and predicted Boolean states is visualized in Figure 4.

For the monocyte lineage, our model correctly predicts the major monocytic players C/EBP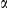 and PU.1 as well as the PU.1-cofactor cJun to be upregulated. Furthermore, the counter-antagonistic activity of EgrNab and Gfi-1 is fully captured, as EgrNab is upregulated and Gfi-1 is strongly suppressed. As expected, we observe a flipped pattern for these two factors in the granulocytic lineage. In the erythroid lineage, all GM players, including the primary fate determinants C/EBP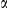 and PU.1, are strongly repressed in both computational model and measured data. GATA-1 as the central MegE player is strongly expressed along with its cofactor FOG-1. SCL follows the expression patterns of PU.1 and GATA-1. This is in accordance with findings that SCL plays a role in HSCs, but is absent throughout non-erythroid maturating cell types [52]. The erythrocyte-specific transcription factor EKLF is upregulated and represses the activity of Fli-1, its counter-antagonist in the megakaryocyte lineage.

Our model is in good agreement with the microarray profiles, except for GATA-2 in the MegE lineage. GATA-2 cannot be considered strongly downregulated in the mRNA expression data of megakaryocyte and erythrocyte progenitor cells. However, the downregulation of GATA-2 is explicitly delayed via the inhibitory coherent feed-forward loop discussed above (see Figure 2A). This view is corroborated by the strong downregulation of GATA-2 in maturated erythrocytes, a late stage of the differentiation process (cf. Text S2). In conclusion, the literature-derived regulatory model is capable of reproducing known molecular phenotypes in terms of mRNA expression profiles.

### *In silico* perturbations

**Figure 5:**
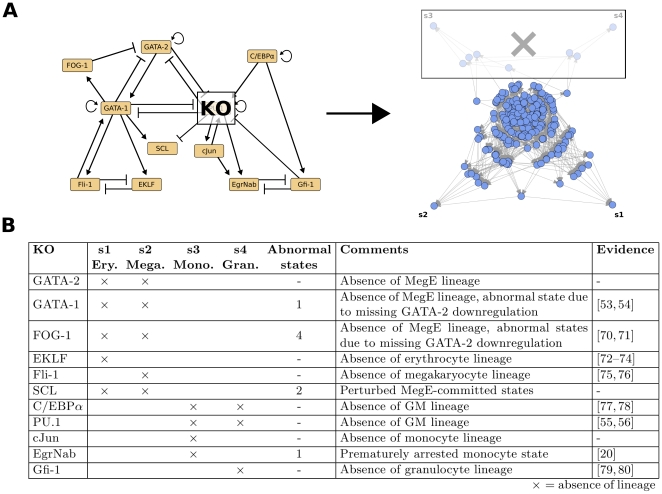
In silico knockout experiments. (**A**) Example case. When setting the expression value of PU.1 to zero in the model (left), a specific set of states becomes unreachable in the state space (right). In this case, these states correspond to the differentiation trajectories and attractors of the granulocytes and macrophage lineages. That is, functionally, we predict all myeloid progenitor cells to differentiate into the MegE lineage upon PU.1 knockout. (**B**) Knockout effects for all 11 players in our model. For each knockout we determined which of the original 4 attractors are still reachable and whether new attractors emerged. The ‘Comments’ column contains brief descriptions of the predicted effects on the differentiation process. In the ‘Evidence’ column we list publications that confirm the predictions of the respective *in silico* knockout [20], [53]–[56], [70]–[80].

A reasonable *in silico* implementation of a biological system provides the possibility to attempt system perturbations with computational effort only. This allows us to cross-validate our model with perturbation experiments independent from the studies used to construct its regulatory logics (Table 1). Both nodes and edges of the myeloid differentiation network can be altered. The knockdown of a regulatory player (Figure 5A) could be caused, for example, by the destruction its regulatory promoter sequences, coding sequence mutations that render the gene product nonfunctional or interference by small RNAs. Player overexpression, on the other hand, is experimentally implemented by ectopic transcription factor expression with, e.g., an inducible construct. Regulatory interaction knockouts finally correspond to specific mutations that affect, for instance, the binding affinity of a transcription factor to the promoter region of its target gene. We performed systematic player and interaction perturbations for our regulatory network by altering the equations in the Boolean model (see Methods). To evaluate the consequences of our *in silico* perturbation experiments we again determined the steady states of the resulting state spaces. For each perturbation we checked how many of the four original attractors, corresponding to the four blood cell lineages, are still present and whether new steady states appear.

The effects of most *in silico* player knockouts can be confirmed by reported phenotypes (see Figure 5B). For example, the knockouts of PU.1 and GATA-1 entirely deplete the GM and MegE lineages, respectively [53]–[56]. However, for a few of cases no experimental evidence exists so far. In these cases, our model can be used to predict the altered differentiation dynamics. (i) We identify a fundamental role of GATA-2 for MegE differentiation. Previous studies only detected lethal effects of GATA-2-deficiency in early hematopoietic stem cells and no effects during late, terminal differentiation [57]. Our model complements this view and predicts a loss of the MegE lineage after GATA-2 knockout. (ii) Since SCL has no active regulatory role in the context of our model, we observe no effect on the remaining players and thus no change in the overall number of attractors (the loss of the attractors s1 and s2 in Figure 5B is balanced by two new attractors). Therefore we assume SCL not to have a pivotal role during myeloid differentiation, a prediction that has to be confirmed with e.g. a conditional knockout experiment. (iii) We find the PU.1 cofactor cJun to be specifically required for the monocyte lineage, firstly because it is strongly repressed during granulopoiesis (see Figure 4), and secondly because we see no effect on the granulocytic lineage in the knockout experiment. Moreover, we analyzed the effects of regulatory interaction knockouts for all 28 interactions in the model (Text S3).

Analogously to the player knockouts we performed player overexpression in the early multipotent state and in the four stable states to infer the potential of lineage reprogramming and transdifferentiation. In accordance with the literature, for instance, GATA-1 and PU.1 instruct differentiation into their respective lineages when overexpressed at an early, multipotent state [58]–[60]. Interestingly, we predict GATA-1 to be capable of transdifferentiating a committed GM cell into the MegE lineage, as demonstrated by [61], [62]. A detailed discussion along with implications for the myeloid differentiation process can be found in Text S4.

## Discussion

In this contribution we presented a regulatory network driving differentiation of murine common myeloid progenitors into megakaryocytes, erythrocytes, granulocytes and monocytes. From a thorough study of the existing literature, we constructed a meso-scale Boolean model comprising 11 transcription factors and 28 regulatory interactions. We included only players and interactions that were reported in the particular context of myeloid differentiation and for the respective cell types. Locally, our gene-regulatory network reveals a modular structure composed of well-known functional motifs, like mutual inhibitory switches, feed-forward loops and negative feedback loops. We examplarily demonstrated the local dynamics and functional implications of two subgraphs in the network, one which induces a *one-way street* effect during MegE differentiation, and a second subgraph that shows asymmetrical activation of the granulocyte vs. monocyte switch. Globally, our Boolean model induces a hierarchical state space. That is, the respective opposing lineages are excluded once the differentiating cell is committed to one of the primary lineages (GM and MegE) or, subsequently, to one of the monopotent secondary lineages. The validity of our model was confirmed by explicit comparison of the attractor states with microarray expression profiles from previously published studies. Moreover, we performed *in silico* knockouts of both players and regulatory interactions as well as player overexpression, compared the model results with phenotypes from the literature and derived new hypotheses. Certainly, intermediate expression levels, e.g. found in early lineage priming processes [1], cannot be captured by the Boolean approach. However, without any parametrization, our model is able to properly describe the potential of common myeloid progenitors to hierarchically differentiate into four myeloid lineages.

Our study leads to a number of predictions of potential biological relevance. On the level of transcription factor regulation, we predict an inhibition from the MegE lineage onto the central GM activator C/EBP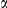. While it is evident from expression profiles that C/EBP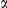 is sharply downregulated once the cell is committed to the MegE lineage (see Figure 4B), no inhibitory regulator of this factor has been identified yet. Our model proposed three players as potential regulators: GATA-1, FOG-1, or SCL. Hints about an indirect inhibition, although in different cell types and differentiation context, exist: In Landry et al. [63], RUNX1 has been identified as a direct target of SCL in the yolk sac, while Tokita et al. [64] showed that the leukemia-inducing fusion protein RUNX1/EVI1 inhibits C/EBP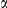 function in LG-3 cells. This regulatory cascade has not been included into our model since the interaction evidences refer differentiation contexts beyond the scope of our model – however, it constitutes a hypothetical interaction that might be worth testing experimentally.

Our model predicts a fifth stable state, which resembles the monocytic lineage profile (PU.1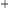, cJun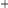, EgrNab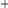) but lacks expression of the early myeloid transcription factor C/EBP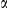. According to this stable state, PU.1 should be able to sustain its own expression even without the presence of C/EBP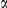. Since we assume C/EBP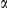 to be the primary activator of PU.1, such an expression pattern (PU.1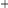, C/EBP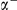) will not occur during physiological hematopoietic differentiation. However, this state might occur due to pathological alteration of the regulatory wiring during disease development.

The model can be used to outline strategies for the forced differentiation and reprogramming of progenitor cells within the myeloid differentiation tree [1], [4], [65], [66]. Our overexpression analysis of the model state space allows for the identification of possible trajectories from one cellular state to another. As an example, consider the conversion of erythrocytes to megakaryocytes. The mutual antagonism of Fli-1 and EKLF determines the final cell fate, and thus, overexpression of the respective antagonist allows to switch the cell from one fate to the other. Interestingly, the lineage reprogramming of both monocytes and granulocytes to the MegE lineage is accomplished by the overexpression of just one factor, GATA-1 or GATA-2. This recapitulates the effect of ectopic GATA-1 overexpression described in [61], [62]. A transdifferentiation of the MegE lineage to the GM side is more intricate since here the coordinated overexpression of C/EBP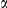 and PU.1 is required.

Currently, our differentiation model describes the core machinery that steers a myeloid progenitor into one of the myeloid lineages, but does not provide an explanation for why a certain decision takes places. To this end, the model could be extended and refined in a number of ways. Most importantly, cytokine signaling has been shown to be a major instructor of lineage decisions [67]. The inclusion of these factors and, more generally, signal transduction pathways, cell-cell signaling [68] and communication with the stem cell niche should provide insights into the mechanisms that give rise to a constant ratio of various blood cells. Furthermore, the incorporation of other regulatory species with impact on hematopoiesis, like microRNAs (see [69] for an extensive review) would allow for a more detailed picture of the differentiation process.

Taken together, we assembled a computational model of myeloid development in mouse which is in accordance with previously acquired molecular data. The hierarchical structure of our transcription factor network directly induces a hierarchical state space and thus typifies general principles of stem cell differentiation. Ultimately, the transfer of the murine model to human hematopoiesis might lay the groundwork for the diagnosis and possibly the treatment of severe disorders in the blood system.

## Materials and Methods

### mRNA expression datasets

We downloaded two mRNA expression studies of hematopoietic progenitor cells from the ArrayExpress database (http://www.ebi.ac.uk/microarray-as/ae/): (i) A study investigating self-renewal and differentiation mechanisms by Chambers et al. [50]. The experiment includes measurements of long-term hematopoietic stem cells and mature blood cells, including erythrocytes, granulocytes, monocytes, B cells, T cells and natural killer cells. For our work we only used the erythrocyte, granulocyte and monocyte data. ArrayExpress ID: E-GEOD-6506. (ii) Pronk et al. [51] attempted to elucidate differential expression patterns during myeloerythroid differentiation by mRNA profiling. Megakaryocyte-erythrocyte progenitors (MEPs), granulocyte-macrophage progenitors (GMPs), common lymphoid progenitors (CLPs) as well as monopotent megakaryocyte and erythrocyte progenitors were measured. For our work we investigated the profiles of the megakaryocyte and erythrocyte progenitors. ArrayExpress ID: E-GEOD-8407.

Both datasets are based on the Affymetrix GeneChip Mouse Genome 430 2.0 and were used as downloaded from the ArrayExpress database (MAS5-normalized and logarithmized to the base of 2). For each nucleotide probe, the median over all replicates was calculated as an average value of expression of the respective probe. Since we cannot distinguish between different variants of a gene transcript, multiple probes for a single gene locus were averaged by median calculation subsequently. A complete list of all probes used in our study is provided in Text S5. For a better comparison of measured and Boolean states, we linearly scaled the expression values in each study between 0 (minimal expression) and 1 (maximal expression). Genes that shows a total maximum fold change smaller than 2 were excluded from the respective experiment.

### Model perturbations

We performed three types of perturbations on our Boolean model: (1) Factor knockouts, (2) factor overexpression, and (3) interaction knockouts. A regulatory factor is knocked out by simply setting its value in the respective Boolean equation to zero or ‘off’. Analogously, overexpression is modeled by setting a factor's value to one or ‘on’. An interaction knockout, on the other hand, can be modeled by setting the regulator to zero in the Boolean equation of its regulated target. For instance, the knockdown of the activatory influence of B towards A in
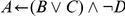
yields a perturbed Boolean equation which reads
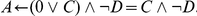



## Supporting Information

Text S1 Detailed description of regulatory interactions
(PDF)

Text S2 Downregulation of GATA-2 during erythrocyte differentiation
(PDF)

Text S3 Regulatory interaction knockouts
(PDF)

Text S4 Overexpression experiments
(PDF)

Text S5 mRNA probes
(PDF)
