# Supplementary material for: Hierarchical Differentiation of Myeloid Progenitors Is Encoded in the Transcription Factor Network
Source: PLoS One. 2011 Aug 10;6(8):e22649. doi: 10.1371/journal.pone.0022649 (PMC3154193; doi:10.1371/journal.pone.0022649)
Supplement: Text S1 — Detailed description of regulatory interactions (PDF) [file pone.0022649.s001.pdf]

Supporting Information S1
Detailed description of regulatory interactions

Reference: Krumsiek, Marr *et al.*, Hierarchical differentiation of myeloid progenitors is encoded in the transcription factor network, *PLoS ONE*.

Notation: $\wedge$ = AND, $\vee$ = OR, $\overline{\text{X}}$ = not X

- GATA-2 = GATA-2 $\wedge$ ($\overline{\text{GATA-1} \wedge \text{FOG-1}}$) $\wedge$ $\overline{\text{PU.1}}$

  As described above, GATA-2 is an early hematopoietic transcription factor that directs differentiation into the MegE lineage by activating GATA-1. GATA-1 and FOG-1 in turn synergize to downregulate the activatory function of GATA-2 on its own promoter, pushing the differentiation process towards maturated blood cells [1, 2]. As both factors are required to exhibit this repressory mechanism, we implemented their influence towards GATA-2 with a Boolean AND gate.

- GATA-1 = (GATA-1 $\vee$ GATA-2 $\vee$ Fli-1) $\wedge$ $\overline{\text{PU.1}}$

  GATA-2 is expressed in immature hematopoietic progenitor cells and activates GATA-1 to drive differentiation towards the MegE lineage [1]. GATA-1, in turn, activates its own expression by direct interaction of a GATA-1 homodimer protein complex with the GATA-1 proximal promoter [3, 4, 5]. Starck et al. [6] found that the GATA-1 downstream factor Fli-1 enhances the stimulatory activity of GATA-1 on GATA-1-responsive promoters. We assume this to have a positive effect on the autoregulation of GATA-1, making Fli-1 an indirect GATA-1 transcriptional activator. Finally, PU.1 and GATA-1 mutually inhibit each other's promoter activity in both mice [7, 8] and human [9] cells.

- FOG-1 = GATA-1

  The transcription factor FOG-1 acts as a cofactor of GATA-1 and is necessary for megakaryocytic and erythroid differentiation [10, 11]. Iwasaki et al. [12] demonstrated that GATA-1 upregulates FOG-1 expression in lymphoid or granulocyte/megakaryocyte progenitor cells.

- EKLF = GATA-1 $\wedge$ $\overline{\text{Fli-1}}$
  Fli-1 = GATA-1 $\wedge$ $\overline{\text{EKLF}}$

  GATA-1 has been shown to be crucial for the expression of the erythrocyte lineage factor EKLF [13]. Moreover, there is evidence for the regulation of the megakaryocyte transcription factor Fli-1 by GATA-1 [14]. Studies of the dependence of GATA-1 on its cofactor showed that FOG-1 is dispensable for the induction of EKLF by GATA-1 [15, 16]. In addition, EKLF and Fli-1 repress each other's transcriptional activity on erythrocyte- and megakaryocyte-specific promoters, respectively [6]. This mutual inhibitory circuit creates the decision switch in the MegE lineage.

- SCL = GATA-1 $\wedge$ $\overline{\text{PU.1}}$

  SCL is a central hematopoietic player required for both primitive and definitive hematopoiesis [17, 18]. However, sustaining the expression of SCL requires different activators during the differentiation process. GATA-1 has been shown to specifically target the SCL promoter during erythroid differentiation [19]. PU.1 inhibits the expression of SCL [20] in the same context. Thus, the SCL player in our model solely represents the SCL protein which is active in the MegE lineage.

- C/EBPα = C/EBPα $\wedge$ ($\overline{\text{GATA-1} \wedge \text{FOG-1} \wedge \text{SCL}}$)

  The major granulocyte/monocyte transcription factor C/EBPα has been shown to be a strong promoter of its own gene [21]. However, to the best of our knowledge, there is no experimental evidence for upstream regulatory factors of C/EBPα (literature research and personal communication). However, the factor is strongly downregulated during megakaryocyte/erythrocyte development [22] and thus requires one of the factors from the opposing lineage to be a direct or indirect inhibitor of C/EBPα. In our model the inhibition could be exhibited by, for instance, GATA-1, SCL or FOG-1. For the model derivation process, we require all three of these MegE factors to be active to constitute C/EBPα inhibition.

- $\text{PU.1} = (\text{C/EBP}\alpha \vee \text{PU.1}) \wedge (\overline{\text{GATA-1} \vee \text{GATA-2}})$

  C/EBPα is known to be a major inducer of PU.1 during GM development and drives the CMP to GMP transition. It directly binds to a distal cis-regulatory element upstream of the PU.1 promoter to stimulate PU.1 mRNA transcription [23, 24]. PU.1 has been shown to autoregulate its expression in murine and human myeloid cells [25]. An autoregulatory loop mediated by an upstream regulatory element of the PU.1 promoter has been postulated by Okuno et al. [25]. In addition, as described above, PU.1 and the GATA factors mutually antagonize each other's promoter activity. The binding of GATA-1 and GATA-2 proteins to the PU.1 promoter and subsequent repression have been shown by Chou et al. [7].

- $\text{cJun} = \text{PU.1} \wedge \overline{\text{Gfi-1}}$

  Steidl et al. [26] demonstrated the necessity of PU.1 for the expression of cJun in preleukemic mouse hematopoietic stem cells. It is to be noted that no mechanistic explanation for this regulatory interaction is provided in the study. Dahl et al. [27] reported that the granulocytic transcription factor Gfi-1 antagonizes the transcriptional activity of PU.1. This interaction does not reflect a change of the expression state of PU.1 but rather an alteration of its effect as an activator of its target genes. Thus, we included Gfi-1 in the equation of cJun as a repressor of the activation by PU.1. In human hematopoietic differentiation, cJun is known to positively autoregulate its own promoter [28]. However, since no comparable study has been published for murine hematopoiesis, we did not include such an autoregulatory loop of cJun in the model.

- $\text{EgrNab} = (\text{PU.1} \wedge \text{cJun}) \wedge \overline{\text{Gfi-1}}$

  We integrate the monocytic transcription factors Egr-1, Egr-2 and Nab-2 into a combined pseudo-player EgrNab as proposed by Laslo et al. [29]. The transcriptional action of PU.1 has been shown to rely on the cofactor cJun, both proteins constituting a heterodimeric protein complex PU.1:cJun [30]. Thus, we subsequently model their regulation with a logical AND. Laslo et al. [29] proposed a mutual antagonism between EgrNab and Gfi-1 based on knockdown and overexpression experiments. Note that the antagonistic effect of Gfi-1 on EgrNab is also explainable as an effect of the repressive influence of Gfi-1 on PU.1-dependent transcription (cf. cJun above).

- $\text{Gfi-1} = \text{C/EBP}\alpha \wedge \overline{\text{EgrNab}}$

  Laslo et al. [29] proposed an activatory influence of C/EBPα towards Gfi-1 based on both phenomenological observations and subsequent modeling approaches. In addition, as mentioned before, EgrNab and Gfi-1 constitute a mutual antagonistic regulatory circuit.

References

[1] Ohneda, K. and Yamamoto, M. Roles of hematopoietic transcription factors gata-1 and gata-2 in the development of red blood cell lineage. *Acta Haematol*, 108(4):237–245, 2002.

[2] Grass, J.A., Boyer, M.E., Pal, S., Wu, J., Weiss, M.J., and Bresnick, E.H. Gata-1-dependent transcriptional repression of gata-2 via disruption of positive autoregulation and domain-wide chromatin remodeling. *Proc Natl Acad Sci U S A*, 100(15):8811–8816, 2003.

[3] Tsai, S.F., Strauss, E., and Orkin, S.H. Functional analysis and in vivo footprinting implicate the erythroid transcription factor gata-1 as a positive regulator of its own promoter. *Genes Dev*, 5(6):919–931, 1991.

[4] Orkin, S.H. Gata-binding transcription factors in hematopoietic cells. *Blood*, 80(3):575–581, 1992.

[5] Trainor, C.D., Omichinski, J.G., Vandergon, T.L., Gronenborn, A.M., Clore, G.M., and Felsenfeld, G. A palindromic regulatory site within vertebrate gata-1 promoters requires both zinc fingers of the gata-1 dna-binding domain for high-affinity interaction. *Mol Cell Biol*, 16(5):2238–2247, 1996.

[6] Starck, J., Cohet, N., Gonnet, C., Sarrazin, S., Doubeikovskaia, Z., Doubeikovski, A., Verger, A., Duterque-Coquillaud, M., and Morle, F. Functional cross-antagonism between transcription factors fli-1 and eklf. *Mol Cell Biol*, 23(4):1390–1402, 2003.

[7] Chou, S.T., Khandros, E., Bailey, L.C., Nichols, K.E., Vakoc, C.R., Yao, Y., Huang, Z., Crispino, J.D., Hardison, R.C., Blobel, G.A., and Weiss, M.J. Graded repression of pu.1/sfpi1 gene transcription by gata factors regulates hematopoietic cell fate. *Blood*, 114(5):983–994, 2009.

[8] Rekhtman, N., Radparvar, F., Evans, T., and Skoultchi, A.I. Direct interaction of hematopoietic transcription factors pu.1 and gata-1: functional antagonism in erythroid cells. *Genes Dev*, 13(11):1398–1411, 1999.

[9] Zhang, P., Behre, G., Pan, J., Iwama, A., Wara-Aswapati, N., Radomska, H.S., Auron, P.E., Tenen, D.G., and Sun, Z. Negative cross-talk between hematopoietic regulators: Gata proteins repress pu.1. *Proc Natl Acad Sci U S A*, 96(15):8705–8710, 1999.

[10] Tsang, A.P., Visvader, J.E., Turner, C.A., Fujiwara, Y., Yu, C., Weiss, M.J., Crossley, M., and Orkin, S.H. Fog, a multitype zinc finger protein, acts as a cofactor for transcription factor gata-1 in erythroid and megakaryocytic differentiation. *Cell*, 90(1):109–119, 1997.

[11] Tsang, A.P., Fujiwara, Y., Hom, D.B., and Orkin, S.H. Failure of megakaryopoiesis and arrested erythropoiesis in mice lacking the gata-1 transcriptional cofactor fog. *Genes Dev*, 12(8):1176–1188, 1998.

[12] Iwasaki, H., Mizuno, S., Wells, R.A., Cantor, A.B., Watanabe, S., and Akashi, K. Gata-1 converts lymphoid and myelomonocytic progenitors into the megakaryocyte/erythrocyte lineages. *Immunity*, 19(3):451–462, 2003.

[13] Crossley, M., Tsang, A.P., Bieker, J.J., and Orkin, S.H. Regulation of the erythroid kruppel-like factor (eklf) gene promoter by the erythroid transcription factor gata-1. *J Biol Chem*, 269(22):15440–15444, 1994.

[14] Barbeau, B., Barat, C., Bergeron, D., and Rassart, E. The gata-1 and spi-1 transcriptional factors bind to a gata/ebs dual element in the fli-1 exon 1. *Oncogene*, 18(40):5535–5545, 1999.

[15] Crispino, J.D., Lodish, M.B., MacKay, J.P., and Orkin, S.H. Use of altered specificity mutants to probe a specific protein-protein interaction in differentiation: the gata-1:fog complex. *Mol Cell*, 3(2):219–228, 1999.

[16] Letting, D.L., Chen, Y.Y., Rakowski, C., Reedy, S., and Blobel, G.A. Context-dependent regulation of gata-1 by friend of gata-1. *Proc Natl Acad Sci U S A*, 101(2):476–481, 2004.

[17] Orkin, S.H. and Zon, L.I. Hematopoiesis: an evolving paradigm for stem cell biology. *Cell*, 132(4):631–644, 2008.

[18] Pimanda, J.E., Ottersbach, K., Knezevic, K., Kinston, S., Chan, W.Y.I., Wilson, N.K., Landry, J.R., Wood, A.D., Kolb-Kokocinski, A., Green, A.R., Tannahill, D., Lacaud, G., Kouskoff, V., and Göttgens, B. Gata2, fli1, and scl form a recursively wired gene-regulatory circuit during early hematopoietic development. *Proc Natl Acad Sci U S A*, 104(45):17692–17697, 2007.

[19] Bockamp, E.O., McLaughlin, F., Murrell, A.M., Göttgens, B., Robb, L., Begley, C.G., and Green, A.R. Lineage-restricted regulation of the murine scl/tal-1 promoter. *Blood*, 86(4):1502–1514, 1995.

[20] Clech, M.L., Chalhoub, E., Dohet, C., Roure, V., Fichelson, S., Moreau-Gachelin, F., and Mathieu, D. Pu.1/spi-1 binds to the human tal-1 silencer to mediate its activity. *J Mol Biol*, 355(1):9–19, 2006.

[21] Legraverend, C., Antonson, P., Flodby, P., and Xanthopoulos, K.G. High level activity of the mouse ccaat/enhancer binding protein (c/ebp alpha) gene promoter involves autoregulation and several ubiquitous transcription factors. *Nucleic Acids Res*, 21(8):1735–1742, 1993.

[22] Laiosa, C.V., Stadtfeld, M., and Graf, T. Determinants of lymphoid-myeloid lineage diversification. *Annu Rev Immunol*, 24:705–738, 2006.

[23] Friedman, A.D. C/ebpalpha induces pu.1 and interacts with ap-1 and nf-kappab to regulate myeloid development. *Blood Cells Mol Dis*, 39(3):340–343, 2007.

[24] Yeamans, C., Wang, D., Paz-Priel, I., Torbett, B.E., Tenen, D.G., and Friedman, A.D. C/ebpalpha binds and activates the pu.1 distal enhancer to induce monocyte lineage commitment. *Blood*, 110(9):3136–3142, 2007.

[25] Okuno, Y., Huang, G., Rosenbauer, F., Evans, E.K., Radomska, H.S., Iwasaki, H., Akashi, K., Moreau-Gachelin, F., Li, Y., Zhang, P., Göttgens, B., and Tenen, D.G. Potential autoregulation of transcription factor pu.1 by an upstream regulatory element. *Mol Cell Biol*, 25(7):2832–2845, 2005.

[26] Steidl, U., Rosenbauer, F., Verhaak, R.G.W., Gu, X., Ebralidze, A., Otu, H.H., Klippel, S., Steidl, C., Bruns, I., Costa, D.B., Wagner, K., Aivado, M., Kobbe, G., Valk, P.J.M., Passegué, E., Libermann, T.A., Delwel, R., and Tenen, D.G. Essential role of jun family transcription factors in pu.1 knockdown-induced leukemic stem cells. *Nat Genet*, 38(11):1269–1277, 2006.

[27] Dahl, R., Iyer, S.R., Owens, K.S., Cuylear, D.D., and Simon, M.C. The transcriptional repressor gfi-1 antagonizes pu.1 activity through protein-protein interaction. *J Biol Chem*, 282(9):6473–6483, 2007.

[28] Angel, P., Hattori, K., Smeal, T., and Karin, M. The jun proto-oncogene is positively autoregulated by its product, jun/ap-1. *Cell*, 55(5):875–885, 1988.

[29] Laslo, P., Spooner, C.J., Warmflash, A., Lancki, D.W., Lee, H.J., Sciammas, R., Gantner, B.N., Dinner, A.R., and Singh, H. Multilineage transcriptional priming and determination of alternate hematopoietic cell fates. *Cell*, 126(4):755–766, 2006.

[30] Behre, G., Whitmarsh, A.J., Coghlan, M.P., Hoang, T., Carpenter, C.L., Zhang, D.E., Davis, R.J., and Tenen, D.G. c-jun is a jnk-independent coactivator of the pu.1 transcription factor. *J Biol Chem*, 274(8):4939–4946, 1999.
