# Supplementary material for: Hierarchical Differentiation of Myeloid Progenitors Is Encoded in the Transcription Factor Network
Source: PLoS One. 2011 Aug 10;6(8):e22649. doi: 10.1371/journal.pone.0022649 (PMC3154193; doi:10.1371/journal.pone.0022649)
Supplement: Text S2 — Downregulation of GATA-2 during erythrocyte differentiation (PDF) [file pone.0022649.s002.pdf]

Supporting Information S2
Downregulation of GATA-2 during erythrocyte differentiation

Reference: Krumsiek, Marr *et al.*, Hierarchical differentiation of myeloid progenitors is encoded in the transcription factor network, *PLoS ONE*.

Our model predicts GATA-2 to be downregulated during erythrocyte and megakaryocyte differentiation. However, in the microarray data for the progenitor cells of both lineages (Pronk et al. [1]), we observe GATA-2 only partially downregulated. By the inspection of mature erythrocytic cells (Chambers et al. [2]), we get further evidence for a stepwise inhibition of GATA-2.

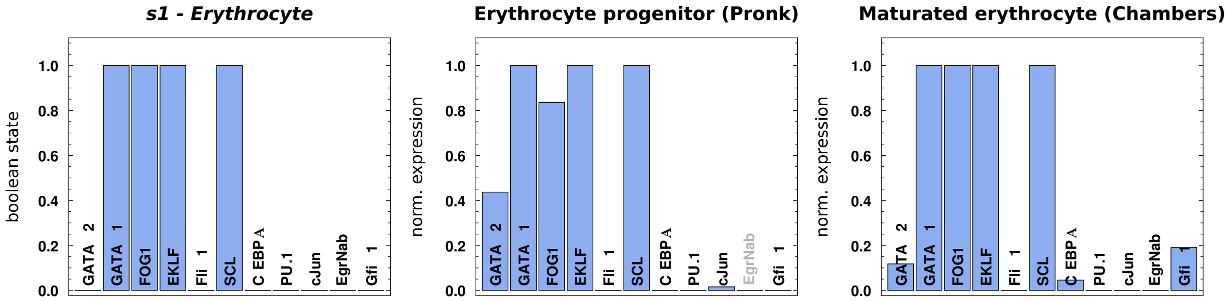

References

[1] Pronk, C.J.H., Rossi, D.J., Månsson, R., Attema, J.L., Norddahl, G.L., Chan, C.K.F., Sigvardsson, M., Weissman, I.L., and Bryder, D. Elucidation of the phenotypic, functional, and molecular topography of a myeloerythroid progenitor cell hierarchy. *Cell Stem Cell*, 1(4):428–442, 2007.

[2] Chambers, S.M., Boles, N.C., Lin, K.Y.K., Tierney, M.P., Bowman, T.V., Bradfute, S.B., Chen, A.J., Merchant, A.A., Sirin, O., Weksberg, D.C., Merchant, M.G., Fisk, C.J., Shaw, C.A., and Goodell, M.A. Hematopoietic fingerprints: an expression database of stem cells and their progeny. *Cell Stem Cell*, 1(5):578–591, 2007.
