# Supplementary material for: Hierarchical Differentiation of Myeloid Progenitors Is Encoded in the Transcription Factor Network
Source: PLoS One. 2011 Aug 10;6(8):e22649. doi: 10.1371/journal.pone.0022649 (PMC3154193; doi:10.1371/journal.pone.0022649)
Supplement: Text S3 — Regulatory interaction knockouts (PDF) [file pone.0022649.s003.pdf]

Supporting Information S3
Regulatory interaction knockouts

Reference: Krumsiek, Marr *et al.*, Hierarchical differentiation of myeloid progenitors is encoded in the transcription factor network, *PLoS ONE*.

The investigation of interaction knockouts yields further insights into the interdependencies and kinetics of transcription factor activation in our myeloid network. Apart from trivial effects, like the knockout of complete lineages by interfering with major activation mechanisms (e.g. from GATA-1 towards EKLF and Fli-1) we will discuss a few cases of special interest in the following. (i) PU.1 does not require its autoregulatory activity during normal differentiation in our model. Only for the hypothetical fifth, PU.1-dependent monocyte state, the self-activation of PU.1 becomes essential. (ii) We observe a mutual compensation of GATA factors for the inhibition of PU.1. Both GATA-1 and GATA-2 can independently repress the PU.1 promoter, ensuring inhibition of the GM lineage at all stages of MegE development, which is in accordance with results from Chou et al. [1]. Similarly, we predict a redundant inhibition of the GATA factors by PU.1, that is each of the inhibitory interactions alone is sufficient to repress the MegE lineage during GM differentiation. (iii) Several asymmetries can be observed for interaction knockout effects in the GM lineage. While the inhibition of Gfi-1 by EgrNab is essential to stabilize the monocyte lineage, the reciprocal repression of EgrNab by Gfi-1 can be compensated for, since Gfi-1 also inhibits the monocyte factor cJun. This dynamical behavior demonstrates system redundancy not detectable on the level of pairwise regulatory interactions alone. (iv) Due to the upregulation of GATA-1 by its downstream target Fli-1 via a positive feedback loop, GATA-1's autoregulatory activity does not appear to be essential once the cell is committed to the megakaryocytic lineage.

**Interaction**	**s1**	**s2**	**s3**	**s4**	**New**	**Comments**
C/EBP$\alpha \overset{+}{\nrightarrow}$ C/EBPα			$\times$	$\times$	-	Absence of GM lineage
GATA-1 $\overset{-}{\nrightarrow}$ C/EBPα	$\times$	$\times$			2	Absence of MegE lineage, no PU.1-dependent state since C/EBPα is not inhibited
C/EBP$\alpha \overset{+}{\nrightarrow}$ PU.1					-	We assume PU.1 to be already active at the beginning of the simulation; if this is not the case, the GM lineage will be absent
PU.1 $\overset{+}{\nrightarrow}$ PU.1					-	Only required without C/EBPα for PU.1-dependent state
GATA-1 $\overset{-}{\nrightarrow}$ PU.1					-	No effect, compensated by GATA-2 inhibition of PU.1
GATA-2 $\overset{-}{\nrightarrow}$ PU.1					-	No effect, compensated by GATA-1 inhibition of PU.1
PU.1 $\overset{+}{\nrightarrow}$ cJun			$\times$		-	Absence of monocyte lineage
Gfi-1 $\overset{-}{\nrightarrow}$ cJun				$\times$	1	Perturbed granulocyte lineage (monocyte factors active)
PU.1 $\overset{+}{\nrightarrow}$ EgrNab			$\times$		1	Absence of monocyte lineage, prematurely arrested
cJun $\overset{+}{\nrightarrow}$ EgrNab			$\times$		1	Absence of monocyte lineage, prematurely arrested
Gfi-1 $\overset{-}{\nrightarrow}$ EgrNab					-	No effect, compensated by downregulation of cJun by Gfi-1
C/EBP$\alpha \overset{+}{\nrightarrow}$ Gfi-1				$\times$	-	Absence of granulocyte lineage
EgrNab $\overset{-}{\nrightarrow}$ Gfi-1			$\times$		-	No monocyte lineage
GATA-1 $\overset{+}{\nrightarrow}$ GATA-1	$\times$				-	No erythrocyte lineage, compensated in megakaryocyte lineage by GATA-1 activation through Fli-1
GATA-2 $\overset{+}{\nrightarrow}$ GATA-1	$\times$	$\times$			1	No MegE lineage, C/EBPα not inhibited
Fli-1 $\overset{+}{\nrightarrow}$ GATA-1					-	No effect, compensated by GATA-1 autoactivation
PU.1 $\overset{-}{\nrightarrow}$ GATA-1					-	Only required for PU.1 dependent state, otherwise compensated by GATA-2 inhibition (upstream of GATA-1)
cJun $\overset{-}{\nrightarrow}$ GATA-1					-	No direct effect, redundant inhibition of MegE lineage (through GATA-2 repression)
GATA-1 $\overset{+}{\nrightarrow}$ FOG-1	$\times$	$\times$			4	Absence of MegE lineage, prematurely arrested states
GATA-2 $\overset{+}{\nrightarrow}$ GATA-2					-	No effect if GATA-1 is activated sufficiently quick
GATA-1 $\overset{-}{\nrightarrow}$ GATA-2					2	Perturbed MegE-committed expression state: GATA-2 not downregulated
FOG-1 $\overset{-}{\nrightarrow}$ GATA-2					2	Perturbed MegE-committed expression state: GATA-2 not downregulated
PU.1 $\overset{-}{\nrightarrow}$ GATA-2					-	No effect, compensated by GATA-1 inhibition of GATA-2
GATA-1 $\overset{+}{\nrightarrow}$ EKLF	$\times$				-	Absence of erythrocyte lineage
Fli-1 $\overset{-}{\nrightarrow}$ EKLF		$\times$			-	Absence of megakaryocyte lineage
GATA-1 $\overset{+}{\nrightarrow}$ Fli-1		$\times$			-	Absence of megakaryocyte lineage
EKLF $\overset{-}{\nrightarrow}$ Fli-1	$\times$				-	Absence of erythrocyte lineage
GATA-1 $\overset{+}{\nrightarrow}$ SCL	$\times$	$\times$			2	Perturbed MegE-committed expression states

Effects of the regulatory interaction knockouts for all 28 interactions in our model. For each knockout we determined which of the original 4 attractors are still reachable and whether new attractors emerged. The 'Comments' column contains brief descriptions of the predicted effects on the differentiation process. $\overset{+}{\nrightarrow}$ = deletion of activation, $\overset{-}{\nrightarrow}$ = deletion of inhibition. Assigned states: s1=erythrocyte, s2=megakaryocyte, s3=monocyte, s4=granulocyte, New=number of new states.

References

[1] Chou, S.T., Khandros, E., Bailey, L.C., Nichols, K.E., Vakoc, C.R., Yao, Y., Huang, Z., Crispino, J.D., Hardison, R.C., Blobel, G.A., and Weiss, M.J. Graded repression of pu.1/sfpi1 gene transcription by gata factors regulates hematopoietic cell fate. *Blood*, 114(5):983–994, 2009.
