# Supplementary material for: Hierarchical Differentiation of Myeloid Progenitors Is Encoded in the Transcription Factor Network
Source: PLoS One. 2011 Aug 10;6(8):e22649. doi: 10.1371/journal.pone.0022649 (PMC3154193; doi:10.1371/journal.pone.0022649)
Supplement: Text S4 — Overexpression experiments (PDF) [file pone.0022649.s004.pdf]

Supporting Information S4
Overexpression experiments

Reference: Krumsiek, Marr *et al.*, Hierarchical differentiation of myeloid progenitors is encoded in the transcription factor network, *PLoS ONE*.

Forced differentiation

In this first analysis we checked how overexpression of one of the factors in the model affects the differentiation dynamics from the early multipotent state (where only GATA-2, C/EBPα, and PU.1 are active), and whether the system is forced to differentiate into a certain lineage. We model overexpression by simply setting the state of the respective factor to a constant value of 1. The following table contains a summary note for each factor as well as the precise steady states the system can reach when overexpressing the factor. Each row corresponds to one steady state.

**Overexpr.**	**C/EBPα**	**PU.1**	**cJun**	**EgrNab**	**Gfi-1**	**GATA-1**	**FOG-1**	**GATA-2**	**EKLF**	**Fli-1**	**SCL**	**Comment**
**C/EBPα**												**no proper differentiation**
	1	1	0	0	1	0	0	0	0	0	0	regular granulo
	1	1	1	1	0	0	0	0	0	0	0	regular mono
	1	0	0	0	1	1	1	0	1	0	1	granulo/ery mix
	1	0	0	0	1	1	1	0	0	1	1	granulo/meg mix
**PU.1**												**forced GM differentiation**
	1	1	0	0	1	0	0	0	0	0	0	regular granulo
	1	1	1	1	0	0	0	0	0	0	0	regular mono
**cJun**												**no forcing effect**
	1	1	1	1	0	0	0	0	0	0	0	regular mono
	1	1	1	0	1	0	0	0	0	0	0	perturbed granulo
	0	0	1	0	0	1	1	0	1	0	1	perturbed ery
	0	0	1	0	0	1	1	0	0	1	1	perturbed meg
**EgrNab**												**no granulo, perturbed MegE**
	1	1	1	1	0	0	0	0	0	0	0	regular mono
	0	0	0	1	0	1	1	0	1	0	1	perturbed ery
	0	0	0	1	0	1	1	0	0	1	1	perturbed meg
**Gfi-1**												**no mono, perturbed MegE**
	1	1	0	0	1	0	0	0	0	0	0	regular granulo
	0	0	0	0	1	1	1	0	1	0	1	perturbed ery
	0	0	0	0	1	1	1	0	0	1	1	perturbed meg
**GATA-1**												**forced MegE differentiation**
	0	0	0	0	0	1	1	0	1	0	1	regular ery
	0	0	0	0	0	1	1	0	0	1	1	regular meg
**FOG-1**												**no forcing effect**
	1	1	0	0	1	0	1	0	0	0	0	perturbed granulo
	1	1	1	1	0	0	1	0	0	0	0	perturbed mono
	0	0	0	0	0	1	1	0	1	0	1	regular ery
	0	0	0	0	0	1	1	0	0	1	1	regular meg
**GATA-2**												**basically, forced MegE diff (+GATA-2)**
	0	0	0	0	0	1	1	1	1	0	1	ery + GATA-2
	0	0	0	0	0	1	1	1	0	1	1	meg + GATA-2
**EKLF**												**no meg, perturbed GM**
	1	1	0	0	1	0	0	0	1	0	0	perturbed granulo
	1	1	1	1	0	0	0	0	1	0	0	perturbed mono
	0	0	0	0	0	1	1	0	1	0	1	regular ery
**Fli-1**												**no ery, perturbed GM**
	1	1	0	0	1	0	0	0	0	1	0	perturbed granulo
	1	1	1	1	0	0	0	0	0	1	0	perturbed mono
	0	0	0	0	0	1	1	0	0	1	1	regular meg
**SCL**												**no effect, despite SCL+**
	1	1	0	0	1	0	0	0	0	0	1	regular granulo + SCL
	1	1	1	1	0	0	0	0	0	0	1	regular mono + SCL
	0	0	0	0	0	1	1	0	1	0	1	regular ery + SCL
	0	0	0	0	0	1	1	0	0	1	1	regular meg + SCL

Interestingly, only GATA-1 and PU.1, the two central players of myeloid lineage decision, are capable of forcing the system into the genuine MegE and GM lineages, respectively. For GATA-1, this is in accordance with published results [1, 2]. In addition, the downstream MegE factor SCL has been shown not to instruct lineage commitment [3], as in our model, where SCL has no downstream factors. Similarly, Iwasaki et al.

[2] showed that 'FOG-1 Does Not Instruct Megakaryocyte/Erythroid Lineage Development', which is compatible with our prediction. To the best of our knowledge, for GATA-2 there is no overexpression phenotype described in the literature. We note that there was a study investigating the expression order of GATA-2 and C/EBPα during lineage choice [4], but there the authors primarily focused on GM and mast cell lineages. In our model, GATA-2 overexpression will drive the system into the MegE lineage, but sustained GATA-2 expression might cause differentiation arrest. On the GM side, PU.1 has been shown to instruct GM commitment in an erythrocyte/megakaryocyte cell line towards the white blood cells [5], which is in accordance with our finding above. In the simulation, the secondary fate determinants of the four mature lineages, EgrNab, Gfi-1, EKLF and Fli-1, are capable of depleting the respective opposite lineage, but cannot constitute fully forced differentiation. Overexpression of cJun generates an abnormal steady state with no clear relevance during normal differentiation.

Lineage reprogramming

In this second analysis we analyzed whether an already differentiated system can be reprogrammed to another lineage by overexpression of one of the factors. Therefore, we here started from each one of the four mature steady states and checked whether a different steady state can be reacher after overexpression. All of these cases are listed in the following table. Each row corresponds to one steady state.

**Overexpr.**	**From**	**Cebpa**	**PU1**	**cJun**	**EgrNab**	**Gfi1**	**GATA1**	**FOG1**	**GATA2**	**EKLF**	**Fli1**	**SCL**	**Comments**
**C/EBPα**													**incomplete reprogramming**
	Ery	1	0	0	0	1	1	1	0	1	0	1	ery → strange new steady state
	Meg	1	0	0	0	1	1	1	0	0	1	1	meg → strange new steady state
**PU.1**													**incomplete reprogramming**
	Ery	0	1	1	1	0	0	0	0	0	0	0	ery → fifth state
	Meg	0	1	1	1	0	0	0	0	0	0	0	meg → fifth state
**cJun**													**no reprogramming**
	Granulo	1	1	1	0	1	0	0	0	0	0	0	defective granu
	Ery	0	0	1	0	0	1	1	0	1	0	1	defective ery
	Meg	0	0	1	0	0	1	1	0	0	1	1	defective meg
**EgrNab**													**reprogramming within GM**
	Granulo	1	1	1	1	0	0	0	0	0	0	0	granu → mono
	Ery	0	0	0	1	0	1	1	0	1	0	1	defective ery
	Meg	0	0	0	1	0	1	1	0	0	1	1	defective meg
**Gfi-1**													**reprogramming within GM**
	Mono	1	1	0	0	1	0	0	0	0	0	0	mono → granu
	Ery	0	0	0	0	1	1	1	0	1	0	1	defective ery
	Meg	0	0	0	0	1	1	1	0	0	1	1	defective meg
**GATA-1**													**full GM→MegE potential**
	Mono	0	0	0	0	0	1	1	0	1	0	1	mono → ery
	Mono	0	0	0	0	0	1	1	0	0	1	1	mono → meg
	Granulo	0	0	0	0	0	1	1	0	1	0	1	granu → ery
	Granulo	0	0	0	0	0	1	1	0	0	1	1	granu → meg
**FOG-1**													**no reprogramming**
	Mono	1	1	1	1	0	0	1	0	0	0	0	defective mono
	Granulo	1	1	0	0	1	0	1	0	0	0	0	defective granu
**GATA-2**													**full GM→MegE potential, but GATA2$^+$**
	Mono	0	0	0	0	0	1	1	1	1	0	1	mono → ery-like with GATA2$^+$
	Mono	0	0	0	0	0	1	1	1	0	1	1	mono → meg-like with GATA2$^+$
	Granulo	0	0	0	0	0	1	1	1	1	0	1	granu → ery-like with GATA2$^+$
	Granulo	0	0	0	0	0	1	1	1	0	1	1	granu → meg-like GATA2$^+$
	Ery	0	0	0	0	0	1	1	1	1	0	1	ery → ery-like with GATA2$^+$
	Meg	0	0	0	0	0	1	1	1	0	1	1	meg → meg-like with GATA2$^+$
**EKLF**													**reprogramming within MegE**
	Mono	1	1	1	1	0	0	0	0	1	0	0	defective mono
	Granulo	1	1	0	0	1	0	0	0	1	0	0	defective granu
	Meg	0	0	0	0	0	1	1	0	1	0	1	meg→ery
**Fli-1**													**reprogramming within MegE**
	Mono	1	1	1	1	0	0	0	0	0	1	0	defective mono
	Granulo	1	1	0	0	1	0	0	0	0	1	0	defective granu
	Ery	0	0	0	0	0	1	1	0	0	1	1	ery→meg
**SCL**													**no reprogramming**
	Mono	1	1	1	1	0	0	0	0	0	0	1	defective mono
	Granulo	1	1	0	0	1	0	0	0	0	0	1	defective granu

Notably, there are only two studies describing transdifferentiation from GM to MegE cells through GATA-1 overexpression [6, 7], which is in accordance with the simulation. All other model phenotypes thus represent predictions of our model. For instance, while GATA-1 overexpression is sufficient to drive cells from committed GM lineages into the MegE lineage, overexpression of PU.1 our C/EBPα alone will not instruct complete transdifferentiation. The downstream secondary fate determinants EgrNab, Gfi-1, EKLF and Fli-1, are capable of reprogramming the respective opposite secondary lineage (e.g. monocytes into granulocytes), but produce aberrant states in the opposing primary lineage. As in the forced differentiation analysis above, FOG-1, cJun and SCL play no roles during reprogramming processes in the model. It is to be noted that FOG-1 has been shown to interact with GATA-1 and GATA-2 in the context reprogramming mast cell into the myeloid lineage [8], which however is outside of the scope of our model.

References

[1] Yamaguchi, Y., Zon, L.I., Ackerman, S.J., Yamamoto, M., and Suda, T. Forced gata-1 expression in the murine myeloid cell line m1: induction of c-mpl expression and megakaryocytic/erythroid differentiation. *Blood*, 91(2):450–457, 1998.

[2] Iwasaki, H., Mizuno, S., Wells, R.A., Cantor, A.B., Watanabe, S., and Akashi, K. Gata-1 converts lymphoid and myelomonocytic progenitors into the megakaryocyte/erythrocyte lineages. *Immunity*, 19(3):451–462, 2003.

[3] Visvader, J.E., Elefanty, A.G., Strasser, A., and Adams, J.M. Gata-1 but not scl induces megakaryocytic differentiation in an early myeloid line. *EMBO J*, 11(12):4557–4564, 1992.

[4] Iwasaki, H., ichi Mizuno, S., Arinobu, Y., Ozawa, H., Mori, Y., Shigematsu, H., Takatsu, K., Tenen, D.G., and Akashi, K. The order of expression of transcription factors directs hierarchical specification of hematopoietic lineages. *Genes Dev*, 20(21):3010–3021, 2006.

[5] Nerlov, C. and Graf, T. Pu.1 induces myeloid lineage commitment in multipotent hematopoietic progenitors. *Genes Dev*, 12(15):2403–2412, 1998.

[6] Kulessa, H., Frampton, J., and Graf, T. Gata-1 reprograms avian myelomonocytic cell lines into eosinophils, thromboblasts, and erythroblasts. *Genes Dev*, 9(10):1250–1262, 1995.

[7] Heyworth, C., Pearson, S., May, G., and Enver, T. Transcription factor-mediated lineage switching reveals plasticity in primary committed progenitor cells. *EMBO J*, 21(14):3770–3781, 2002.

[8] Cantor, A.B., Iwasaki, H., Arinobu, Y., Moran, T.B., Shigematsu, H., Sullivan, M.R., Akashi, K., and Orkin, S.H. Antagonism of fog-1 and gata factors in fate choice for the mast cell lineage. *J Exp Med*, 205(3):611–624, 2008.
