# Supplementary material for: Hierarchical Differentiation of Myeloid Progenitors Is Encoded in the Transcription Factor Network
Source: PLoS One. 2011 Aug 10;6(8):e22649. doi: 10.1371/journal.pone.0022649 (PMC3154193; doi:10.1371/journal.pone.0022649)
Supplement: Text S5 — mRNA probes (PDF) [file pone.0022649.s005.pdf]

Supporting Information S5
mRNA probes

Reference: Krumsiek, Marr *et al.*, Hierarchical differentiation of myeloid progenitors is encoded in the transcription factor network, *PLoS ONE*.

The following table provides a list of mRNA probe IDs on the Affymetrix GeneChip Mouse Genome 430 2.0 according to the MGI database for all gene products used in our study.

**Gene**	**Probe ID(s)**
C/EBPα	4118982_at
Sfpi1 (PU.1)	1418747_at
Jun	1417409_at, 1448694_at
Gfi1	1417679_at
Egr1	1417065_at
Egr2	1427683_at, 1427682_a_at
Nab2	1417930_at
Gata2	1450333_a_at, 1428816_a_at
Gata1	1449232_at
Fog1	1451046_at
EKLF	1418600_at
Fli1	1422024_at
Tal1 (SCL)	1449389_at
